# Splenic re-irradiation for waldenstrőm’s macroglobulinemia

**DOI:** 10.1186/1748-717X-7-58

**Published:** 2012-04-12

**Authors:** Zhou Wei, Yu Yanxia, Xu Ying, Wang Han, Jiang Yuhua

**Affiliations:** 1 Department of Radiotherapy, Cancer Centre, Qilu Hospital, Shandong University, 107 Wenhuaxi Street, Jinan, Shandong, 250012, China; 2 Cancer Centre, the Second Hospital of Shandong University, 247 Beiyuan Street, Jinan, Shandong, 250033, China

**Keywords:** Waldenström’s macroglobulinemia, Lymphoma, Radiotherapy

## Abstract

We report on a case of Waldenström’s macroglobulinemia (WM) treated with splenic re-irradiation. To the best of our knowledge this has not been reported before. A 69-year-old Asian female patient with WM received a three-dimensional conformal radiotherapy, with 24 Gy in 12 treatment fractions in the first stage. She tolerated the treatment well, with a 37% reduction of the monoclonal immunoglobulin, an impalpable spleen, and improved hematological laboratory tests for 4 months. She was then treated with splenic re-irradiation up to 24 Gy for tumor progression. She showed no evidence of progression one year after re-irradiation, with a 55% reduction of the monoclonal immunoglobulin. Our experience demonstrates that splenic irradiation is an effective treatment to control the progression of WM.

## Background

Waldenström’s macroglobulinemia (WM) is a lymphoplasmacytic lymphoma, characteristic of monoclonal immunoglobulin M (IgM) production and bone marrow infiltration [1]. Unfortunately, median survival ranges between 60 and 120 months [2]. Most are symptomatic, such as anemia, leukopenia, splenomegaly, or hepatomegalanemia, and therefore require further treatment to control symptoms [3]. Splenic irradiation (SI) can palliate the pain associated with hypersplenism. Localized radiation of the spleen has been most widely accepted in the management of lymphoproliferative and myeloproliferative disorders [4,5]. However, relatively little has been discussed on the use of SI in the treatment of WM. We report on a case of splenic re-irradiation for treatment of WM progression. To the best of our knowledge this has not been reported before.

## Case presentation

**Figure 1:**
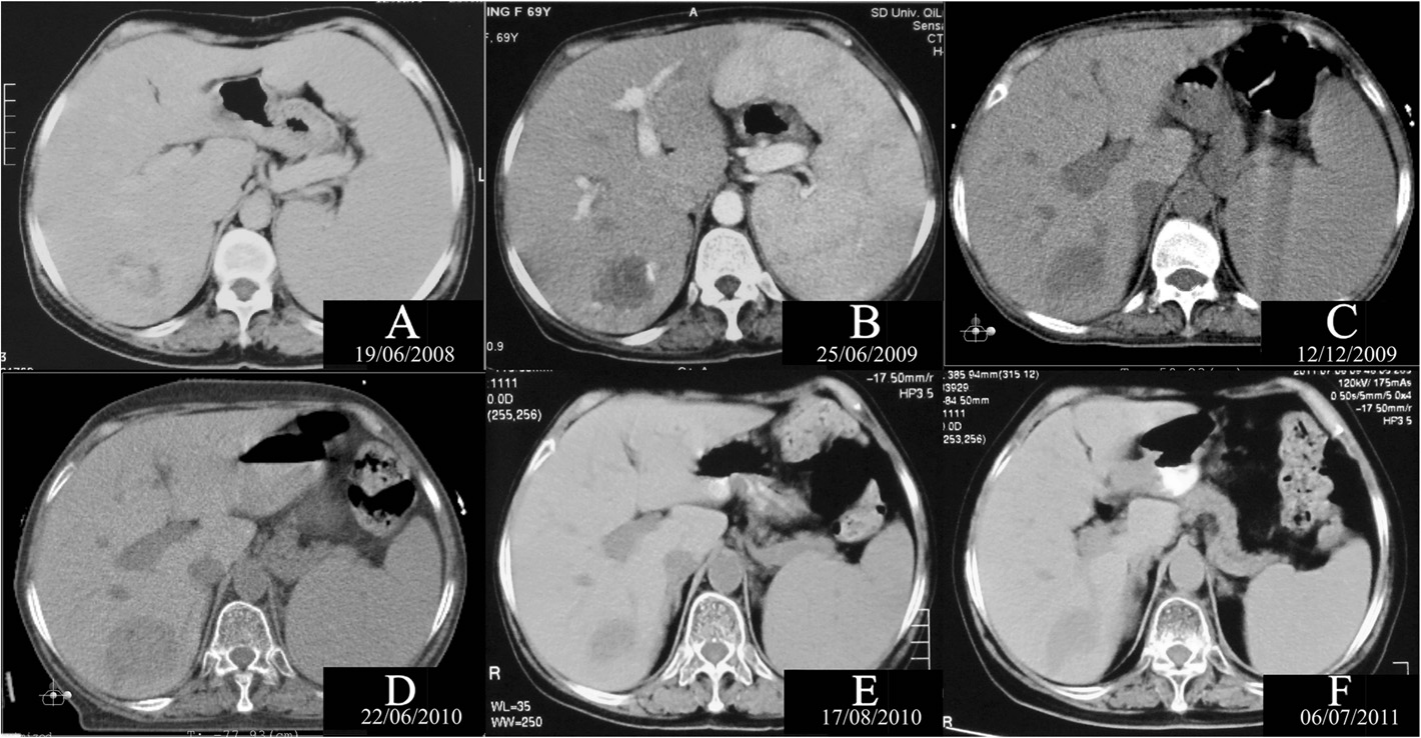
**Sequential computed tomography scan images at the same level. A**, At the diagnosis of the disease (19/06/2008); **B**, Relapsed after chlorambucil and thalidomide (25/06/2009); **C**, Before the first stage of 3D-CRT (12/12/2009); **D**, Before the second stage of 3D-CRT (22/06/2010); **E**, At one month after the second stage of 3D-CRT (17/08/2010); **F**, At one year after the second stage of 3D-CRT (06/07/2011).

A 69-year-old Asian female presented with fatigue and loss of weight (4 kg) in March, 2008. Physical examination revealed massive splenomegaly (4 cm below the left costal margin) (Figure 1A), and hydropericardium confirmed by ultrasound examination and computed tomography (CT). On 19th June, 2008, blood tests found hemoglobin, 101 g/L (110–165 g/L); WBC count, 5.5 × 10^9^/L (4.0–10.0 × 10^9^/L), N 45.0%, L 46.4%; platelet count, 222 × 10^9^/L (100–300 × 10^9^/L). Quantification of serum immunoglobulins revealed the following: IgG 9.9 g/L (7–16 g/L); IgA 0.8 g/L (0.7–4 g/L); IgM 15.8 g/L (0.4–2.3 g/L). Erythrocyte Sedimentation Rate (ESR) is 105 mm/h. Immunofixation electrophoresis of serum showed monoclonal IgM of λ type. Bone marrow aspiration revealed WM. The chest and skeletal radiologic survey revealed no abnormality. The patient was subsequently commenced on chlorambucil successively. After the use of chlorambucil, he showed a 50% reduction of the monoclonal immunoglobulin, a disappearance of lymphoplasmacytic neoplastic cells from the bone marrow, and an impalpable spleen. But she developed persistent profound pancytopenia, and this was therefore stopped. The patient had been under surveillance for 5 months, with no further active treatment. Bone marrow aspiration and biopsy were repeated, which had showed focal hypocellularity without any abnormal cell infiltration until May 2009. At that time, the spleen increased to the level of 7 cm below the costal margin in one month. Further treatment was thalidomide at a starting dose of 200 mg daily with dose escalation to 400 mg. But within 20 days the patient developed transient grade 3 leukopenia and neutropenia plus persistent grade 3 anemia, which led to discontinuation. The spleen was still 7 cm below the costal margin (Figure 1B). In June 2009, she received FC combination therapy (fludarabine 50 mg for 5 days plus cyclophosphamide 800 mg for 1 day intravenously, repeated every 28 days). After one cycle, she showed a 36% reduction of the monoclonal immunoglobulin. Furthermore, the splenomegaly had decreased from 7 cm to 3 cm, below the costal margin by August 2009. However, the patient had persistent grade 3 leukopenia plus persistent grade 3 anemia which lasted for 6 months, and this was therefore stopped. During this period, the patient had herpes in crissum and pneumonia. Although the patient received human granulocyte colony stimulating factors (hG-CSF) and erythropoietin (EPO) repeatedly, anemia and leukopenia relapsed soon.

The patient was then referred to SI in December 2009. At that time, palpable splenomegaly increases to 5 cm inferior to costal margin (Figure 1C). A three-dimensional conformal radiation therapy (3D-CRT) plan was designed. In the first stage, the dose prescribed was 24 Gy in 12 treatment fractions via a Varian 23EX linear accelerator. Three oblique isocentric photon fields (4°, 103° and 179°) of beam quality 10-MV were delivered. Mean dose to left kidney, right kidney were 617 cGy and 264 cGy, respectively. Maximum dose to spinal cord was 716 cGy. The patient had been completely asymptomatic with impalpable spleen, and normal hematological laboratory tests for 4 months. In February 2010, she showed a 37% reduction of the monoclonal immunoglobulin.

**Figure 2:**
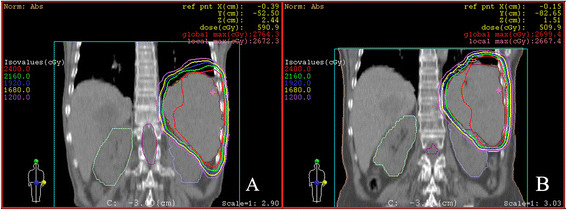
**Transaxial slices representative of isodose distribution of 3D-CRT treatment plan delivered for the whole spleen. A**, In the first stage, isodose curves were produced by 3 oblique isocentric photon fields of beam quality 10-MV; **B**, In the second stage, isodose curves were produced by 5 oblique isocentric photon fields of beam quality 10-MV.

The patient presented complaining of several weeks of generalized weakness and fatigue with pancytopenia in June 2010. Quantification of serum immunoglobulins showed that IgM increased to 8.10 g/L (0.4–2.3 g/L). Physical examinations demonstrated that the spleen was impalpable below the costal margin (Figure 1D). The patient refused to receive chemotherapy or splenectomy, so she was treated with splenic re-irradiation. The second stage consisted of 12 fractions, 2 Gy each, by 5 oblique isocentric photon fields (314°, 3°, 32°, 138°, and 179°) of beam quality 10 MV (Figure 2B). The V18 of kidneys for the sum plan of the two radiation therapies, were 23.2% (left) and 0.0% (right). The V10 of kidneys were 46.3% (left) and 0.0% (right). She tolerated conformal radiotherapy up to 24 Gy well (Figure 1E). Until July, 2011, she has shown no evidence of progression after re-irradiation for one year, with no further treatment (Figure 1F). In July 2011, IgM decreased to 3.65 g/L (0.4–2.3 g/L), a 55% reduction of the monoclonal immunoglobulin as compared with before re-irradiation.

## Discussion and Recommendations

In hematological disorders, splenomegaly is often involved with anemia, leukopenia, as well as thrombocytopenia, which increase the risks of splenectomy. At this time, SI is deemed to be another effective treatment of hypersplenism. However, the effects of SI are usually transient. In chronic lymphocytic leukemia, massive splenomegaly is proved to be related to a shorter therapy-free as well as overall survival [6]. WM progressed in our patient after four months’ completely asymptomatic. The mean duration of remission after palliative spleen irradiation reported in most of the studies was 3 to 6 months, although some cases showed sustained periods of responses [7]. Takemori *et al*. [8] and Cavanna *et al*. [9] reported two cases of WM treated with chemotherapy and SI resulting in a transient remission, while splenectomy achieving a sustained remission for 6 years and 18 years, respectively.

Here, our patient refused to take chemotherapy or splenectomy, so she was treated with splenic re-irradiation for WM progression. She tolerated the treatment of 3D-CRT up to 24 Gy well. With overall doses between 4 and 10 Gy in mostly 1-Gy fractions, symptoms of splenomegaly have been improved in 50–87% of chronic lymphatic leukaemias patients [5]. Although lower dose per fraction and lower total dose radiation have been a standard in the treatment of hematological and myeloproliferative-associated splenomegaly, we advocate higher doses for sustained remission used to patients with WM, especially those who cannot tolerate operation and chemotharapy. SI may remain practicable for the elderly patients, even with total dose of 20 Gy [10]. Re-irradiation for further symptomatic progression is also carried out in other diseases. It was regarded that splenic re-irradiation was feasible without excessive toxicity. We think that our patient can tolerate re-irradiation up to 24 Gy well, partly because of the use of 3D-CRT. The common technique of SI is two parallel opposing anterior-posterior portals encompassing the whole spleen. The use of 3D-CRT is especially beneficial for patients treated with re-irradiation or patients with impaired renal function.

Here, SI reduced spleen size, myelosuppression, as well as high serum IgM level. Based on previous studies on spleen function, we propose that several biological mechanisms are involved in this effect. Firstly, a direct radiation causes the death of circulating lymphoplasmacytoid cells in the spleen. The main radiobiological mechanism of radiation-induced death is mitotic catastrophe, interphase death and terminal growth arrest (senescence) [11]. Secondly, given that the spleen contained abundant IgM-producing lymphocytes, SI impairs the production of monoclonal immunoglobulin. Peripheral blood mononuclear cells are unable to differentiate into immunoglobulin-secreting cells in splenectomized patients [12]. Thirdly, local radiation induces the spleen release of several cytokines, such as TNF-α, INF-γ and IL-2. These cytokines produce an abscopal effect with immunological responses against distant tumor cells [13]. At the same time, SI impairs normal CD8+ T-suppressor cells function [5]. The absence of CD8+ T-suppressor cells enhances anti-tumour response. Fourthly, high dose of radiation may induce spleen fibrosis, which may prevent from recurrent splenomegaly [5].

## Conclusion

Our experience demonstrates that SI is not only a palliative treatment to relieve the pain of splenomegaly, but also an effective treatment to control the progression of WM. In cases of patients who refuse to have a splenectomy for recurrent hypersplenism, splenic re-irradiation may be another treatment. We recommend the use of 3D-CRT in re-irradiation for better renal protection.

## Consent

Written informed consent was obtained from the patient for publication of this case report and any accompanying images. A copy of the written consent is available for review by the Editor-in-Chief of this journal.

## Competing interests

The authors declare that they have no competing interests.

## Authors’ contributions

JYH and ZW participated in the radiotherapy management of this patient and drafted the manuscript. YYX performed the radiotherapy planning, and dosimetric measurements and calculations for this patient. XY and WH contributed the chemotherapy components of the manuscript. All authors read and approved the final manuscript.
